# Sigmoid Venous Thrombosis in JAK2 V617F Mutated Polycythemia Vera

**DOI:** 10.1155/2022/4948115

**Published:** 2022-11-22

**Authors:** Cilomar Martins De Oliveira Filho, Alexander Gavralidis

**Affiliations:** Mass General Brigham, Salem Hospital, Salem, MA, USA

## Abstract

A 60-year-old female presented with headaches, blurry vision, diplopia, and dizziness for six weeks. Her workup revealed an elevated hematocrit, thrombocytosis, high ferritin, and normal erythropoietin. She was diagnosed with polycythemia vera with the JAK2 V617F mutation. The patient underwent magnetic resonance venography, which showed left-sided sigmoid venous thrombosis. She was placed on low-molecular-weight heparin, with a plan to transition to oral anticoagulation after four weeks and repeat imaging in three months to assess for resolution. Thrombotic events may occur in patients with polycythemia vera, and a JAK2 mutation further heightens that risk. Even so, intracranial venous thrombosis is not among the most common events, and it should be kept in the differential for any patient with myeloproliferative neoplasms presenting with new neurological symptoms.

## 1. Introduction

The risk of venous thromboembolism in myeloproliferative neoplasms (MPN), including polycythemia vera, is well established and not uncommon. However, primary venous thromboembolism occurring intracranially is rare. A large retrospective study found a disease-specific frequency of 1.2% for cerebral venous thrombosis (CVT) in patients with polycythemia vera [1]. Women seem to be at higher risk for those events, as are patients with the Janus kinase 2 (JAK2) mutation, and those presenting with leukocytosis [2–4]. Headaches are the leading symptom, but visual disturbances, nausea and vomiting, hemiparesis, seizures, and vertigo have also been reported [1, 5].

Here we report a case of sigmoid venous thrombosis in a patient recently diagnosed with polycythemia vera with the JAK2 V617F mutation, causing headaches and diplopia.

## 2. Case Presentation

A 60-year-old female with a past medical history of ductal carcinoma in situ after lumpectomy and radiation and currently on hormonal therapy with an aromatase inhibitor presented with six weeks of pinpoint frontal headaches, blurry vision, diplopia, and dizziness. Her symptoms worsened over the past two weeks prior to her presentation. Her past medical history is also remarkable for tremors, for which she was seeing a neurologist, obesity, and obstructive sleep apnea on CPAP (continuous positive airway pressure). There are no additional cardiovascular risk factors in her past medical history, and she has never smoked. She is a frequent blood donor, as she claims that her blood counts are always on the higher side.

She started taking topiramate and a trial of indomethacin for the headaches, with only modest improvement. As an outpatient, her hematocrit was found to be elevated at 49.8% (Table 1), and she was referred to a hematologist. Her erythropoietin level was 4.4 mIU/mL (reference range 2.6–18.5). She was found to have a mutation on JAK2 V617F, measured at 16% of total JAK2 DNA (Table 2). Retrospectively, it was discovered that the patient has a sister with polycythemia vera.

The patient was scheduled to initiate phlebotomy treatment with a plan to start hydroxyurea afterwards. She underwent one session of phlebotomy without complications.

On arrival at the hospital, the patient's vital signs were normal. On physical examination, she was awake, alert, and oriented. Extraocular movements were intact with no nystagmus. There was no rest tremor, no dysmetria, and no intention tremor on finger-nose-finger testing. Ambulation was normal. No lymphadenopathy was found. There was no pruritus, no erythromelalgia, no ecchymosis, petechiae, or rash. Respiratory, cardiovascular, and abdominal exams were also unremarkable.

**Figure 1:**
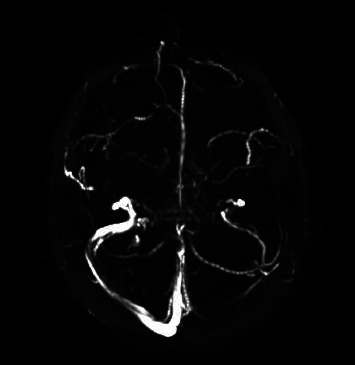
Magnetic resonance venography: *T*1 hyperintensity with lack of enhancement in the lateral aspect of the left proximal sigmoid sinus.

A brain magnetic resonance imaging was ordered and failed to reveal an acute change or finding to explain her symptoms. Consequently, magnetic resonance venography (MRV) was obtained, revealing *T*1 hyperintensity with a lack of enhancement in the lateral aspect of the left proximal sigmoid sinus, compatible with dural venous sinus thrombosis, with likely extension to the left jugular bulb (Figure 1).

The patient was admitted and started on low-molecular-weight heparin. It was suggested that her double vision might have been a partial CN VI palsy secondary to increased intracranial pressure, and a trial of IV acetazolamide (250 mg BID) was given. The patient's headaches and visual disturbances gradually improved, and she was discharged on enoxaparin for four weeks, planning to transition to oral anticoagulation with either direct oral agents or warfarin for three to twelve months, at the discretion of her hematologist. There was also a plan to repeat the MRV in three months to assess its resolution. She had a follow-up two weeks after discharge, and the patient was still experiencing some symptoms, even though they were now milder.

## 3. Discussion

The most common venous thrombotic events in patients with MPNs are deep vein thrombosis, pulmonary embolism, splanchnic vein thrombosis, and superficial thrombophlebitis. Those events are usually found shortly after the diagnosis of either polycythemia vera (PV) or essential thrombocythemia (ET) is made [6]. Patients at risk for thrombosis are those over 60 years of age with a previous history of thrombosis and the JAK2^V617F^ mutation. Additionally, patients with severe thrombocytosis may be at risk for bleeding, as it can be associated with acquired von Willebrand syndrome [7]. Our patient also has a sister with a diagnosis of PV, which raises the possibility of familial MPNs. This is of importance because in patients with either familial PV or ET, the JAK2^V617F^ mutation has been associated with a higher risk of thrombotic events than in those with nonfamilial MPNs, despite bearing a similar prognosis [8]. Finally, in our case, the patient was prescribed an aromatase inhibitor for the treatment of breast cancer. Those agents are not usually associated with thrombotic events, and hence there is a less likely chance that this therapy is associated with the patient's current presentation [9].

### 3.1. Pathogenesis

The pathogenesis leading up to thrombotic events in polycythemia vera is not entirely clear. Blood hyperviscosity is thought to be one of the causes of thrombotic events both in neoplastic and in nonclonal exaggerated erythropoiesis [10]. However, there are likely other implicating factors, given that polycythemia itself would not explain higher rates of thrombosis seen in other MPNs such as essential thrombocythemia. It is postulated that patients with PV present with higher prothrombotic gene expression, including F3, IL10, VEGFA, LDHA, and SLC2A1. These genes were found to correlate positively with a JAK2 mutation in PV [11]. Leukocytosis may also be an implicating cause. The biological plausibility lies in the fact that patients with PV express higher levels of beta-2 integrin, CD11b, and leukocyte alkaline phosphatase, ultimately leading to increased activation of neutrophils. These activated polymorphonuclear cells interact with platelets, impairing normal hemostasis [12]. A meta-analysis confirmed that leukocytosis is associated with increased thrombotic events in PV, but a subgroup analysis revealed that this was true for arterial rather than venous events. Furthermore, the study could not establish a cutoff for white blood cell counts that portended a higher risk for thrombosis [13].

### 3.2. Epidemiology, Clinical Manifestations, and Diagnosis

**Figure 2:**
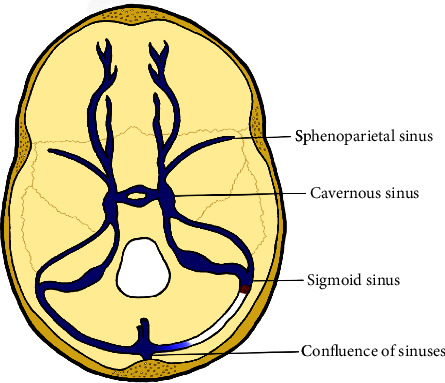
Anatomy of the cerebral venous system and sigmoid sinus thrombosis.

A large observational study found the prevalence of venous thrombotic events, regardless of location, in patients with polycythemia vera to be 9% [2]. Another study identified a prevalence of 1.2% for CVT in patients with PV. The most common sites for cerebral sinus thrombosis in this series of cases were the transverse sinus, the sagittal sinus, and the sigmoid sinus, respectively [1] (Figure 2). Headache and blurry vision are among the leading symptoms; however, nausea and vomiting, diplopia, and seizures have also been reported [1, 5]. CVT should also raise concern for signs and symptoms of intracranial hypertension, such as associated papilledema [14]. Most recent guidelines recommend screening oncologic patients for cerebral venous thrombosis. Furthermore, they state that either magnetic resonance or computed tomography angiography are the tests of choice to diagnose CVT [15].

### 3.3. Management and Prognosis

Few studies address treatment of CVT, specifically in the subgroup of patients with MPNs. In general, low molecular weight heparin (LMWH) seems to be a better option for acute anticoagulation than unfractionated heparin (UFH) when considering adverse events such as major bleeding and thrombotic complications [16]. Heparin should be used in the acute setting, defined as a period short after the diagnosis, when intracranial hypertension and brain herniation may occur [17]. There is no high-quality evidence assessing the duration of anticoagulation. Hence, it is reasonable to maintain oral therapy for a period of 3 to 12 months [15]. The use of direct oral anticoagulants (DOACs) in CVT appears to be a safe choice when compared to coumarins [18, 19]. A large multicenter retrospective study assessed DOACs for treatment of CVT of any cause. It showed no statistically significant difference between DOACs and warfarin regarding outcomes such as recurrence, death, and venous recanalization. It also showed a higher risk of major hemorrhage with warfarin when compared to DOACs [19]. Even though cerebral sinus thrombosis does not seem to affect mortality rates, it might be associated with morbidity as some patients may present persistent neurological symptoms [1]. As for both arterial and venous thromboembolic episodes in the MPN population, observational studies suggest that DOACs seem both effective and safe when compared with vitamin K antagonists [20].

This is a case of cerebral sinus thrombosis in a patient with the JAK2 V617F mutation of polycythemia vera. Even though thrombosis is a well-established complication of myeloproliferative neoplasms, sigmoid thrombosis is not particularly common. The patient presented with headaches and diplopia, signs of intracranial hypertension, and was diagnosed using the gold-standard method, a magnetic resonance venography. Patients with CVT should be treated with anticoagulation for three to 12 months, starting with a heparinoid product in the acute setting and transitioning to oral anticoagulation for the long-term outpatient treatment. This case reinforces that CVT should be kept on the differential for patients with MPNs and new neurological symptoms.

## Figures and Tables

**Table 1:** Baseline complete blood count.

Hemoglobin	16.6 g/dL
Hematocrit	49.8%
RBCs	5.28 M/uL
MCV	94.3 fL
MCH	31.4 pg
MCHC	33.3 g/dL
Platelets	497 K/uL
MPV	10.7 fI
RDW	13.8%
WBC	10.17 K/uL

RBC: red blood cell; MCV: mean corpuscular volume; MCH: mean corpuscular hemoglobin; MCHC: mean corpuscular hemoglobin concentration; MPV: mean platelet volume; RDW: red cell distribution width; WBC: white blood cell.

**Table 2:** Hematology workup.

Ferritin	263 ug/L
Iron	119 ug/dL
Iron saturation	51%
TIBC	234 ug/dL
PT	13 sec
PT-INR	1.0
PTT	26.1 sec
EPO	4.4 mIU/mL
JAK2^V617F^	Positive; 16%

TIBC: total iron binding capacity; PT: prothrombin time; INR: international normalized ratio; PTT: partial thromboplastin time; EPO: erythropoietin; JAK2: Janus kinase 2.

## Data Availability

The data that support the findings of this study are available from the corresponding author upon reasonable request.

## Abbreviations

CVT: Cerebral venous thrombosis
DOAC: Direct oral anticoagulant
JAK2: Janus kinase 2
LMWH: Low-molecular-weight heparin
MPN: Myeloproliferative neoplasm
MRV: Magnetic resonance venography
PV: Polycythemia vera
UFH: Unfractionated heparin.
